# Pollen-derived extracellular vesicles promotes allergic airway inflammation

**DOI:** 10.3389/fimmu.2026.1793997

**Published:** 2026-04-21

**Authors:** Tengze Shang, Junda Li, Yi Ru, Kai Guan

**Affiliations:** Department of Allergy, Peking Union Medical College Hospital, Chinese Academy of Medical Sciences and Peking Union Medical College, Beijing, China

**Keywords:** *Artemisia annua*, asthma, BEAS 2B cells, extracellular vesicles, pollen

## Abstract

**Background:**

Asthma remains a global health burden, affecting over 300 million individuals worldwide, with its pathogenesis involving complex interactions between genetic predisposition and environmental allergens. Pollen is a well-established trigger of allergic asthma. However, the precise mechanisms underlying its allergenic activity remain incompletely understood. Recent advances have highlighted the emerging role of plant-derived extracellular vesicles in immune modulation. Notably, pollen-derived extracellular vesicles (PDEVs) have been identified as carriers of allergenic proteins. Therefore, this study investigates whether pollen contains extracellular vesicles(EVs) and whether these vesicles can induce allergic airway inflammation.

**Methods:**

We isolated extracellular vesicles from *Artemisia annua* pollen using differential centrifugation and sucrose density gradient ultracentrifugation. The biological activity of PDEVs was evaluated *in vitro* using human airway epithelial cells (BEAS-2B) and *in vivo* using a murine asthma model.

**Results:**

PDEVs are nanoscale lipid bilayer structures containing diverse allergenic proteins and exhibiting structural stability. PDEVs induced significantly stronger pro-inflammatory responses compared to pollen supernatant (Sup) *in vitro*. PDEVs enhanced inflammatory cytokine production IL-4, IL-5, IL-13, IL-33 expression, and promoted eosinophilic, neutrophilic infiltration in murine.

**Conclusion:**

Our findings suggest extracellular vesicles present in pollen grains, which may represent a critical mechanism underlying pollen-induced airway inflammation. Targeting PDEVs may offer new therapeutic strategies for allergic airway diseases prevention and treatment.

## 1 Introduction

Asthma is a complex chronic inflammatory disease of the airways with a genetic predisposition, characterized by airway hyperresponsiveness, reversible airflow obstruction, and airway remodeling (1). In recent years, the global prevalence of asthma has continued to rise, currently affecting over 339 million individuals worldwide (2). Allergic asthma, a common asthma subtype, is induced by exogenous allergens (e.g., house dust mites, pollen, fungi, and animal dander) through allergen-specific IgE-mediated airway inflammation. Pollen is a prevalent outdoor aeroallergen (3).

In recent decades, climate change has driven a progressive increase in airborne pollen levels, leading to a corresponding surge in the incidence of pollen-associated asthma (4). Notably, *Artemisia* pollen—a major allergen—has become increasingly prevalent in Asia, Europe, and North America (5). In northern China, it now represents a critical allergen, severely impacting patients’ quality of life. However, pollen grains typically exceed 10 µm in diameter and are largely retained in the upper respiratory tract, rarely reaching the bronchi. While thunderstorm-induced pollen rupture into respirable particles (1–5 μm) is a recognized trigger for asthma exacerbations, this mechanism fails to explain persistent symptoms in non-storm conditions (6). To address this paradox, we explored alternative mechanisms underlying pollen-induced asthma.

Recent advances in extracellular vesicle research have revealed that plant-derived extracellular vesicles contain diverse bioactive cargoes, including proteins, microRNAs, and metabolites, with dimensions typically <1μm in diameter. Studies have demonstrated that plant-derived extracellular vesicles are ubiquitously present in diverse species (7, 8).These nanoscale structures exhibit a unique capacity to translocate across cellular membranes, enabling them to interact with and modulate the function of multiple cell types, including epithelial cells, macrophages, and dendritic cells (9, 10).Current research has predominantly focused on their anti-inflammatory properties, with evidence suggesting therapeutic potential in diseases such as asthma, inflammatory bowel disease, and cancer (11–13). However, limited evidence suggests that plant-derived extracellular vesicles may also exacerbate inflammatory disorders. For instance, study has demonstrated that extracellular vesicles derived from olive pollen (*Olea europaea*) contain major allergenic proteins, including Ole e 1, Ole e 11, and Ole e, suggesting a possible role in allergic asthma (14).

Our study reveals that Artemisia pollen contains extracellular vesicles that induce more pronounced airway inflammation than crude pollen extracts, suggesting that pollen-derived vesicles may represent a previously underappreciated mechanism contributing to pollen-related asthma.

## 2 Methods

### 2.1 Mouse experiments and cell lines

All animal experiments were approved by the Experimental Animal Ethics Committee of Peking Union Medical College Hospital and conducted in accordance with the ethical guidelines established by the Chinese Committee of Animal Welfare. Female BALB/c mice (6–8 weeks of age) were purchased from Charles River Laboratories (Beijing, China).

Human airway epithelial cells (BEAS-2B) were obtained from the American Type Culture Collection (ATCC, USA). All cell lines were maintained in Dulbecco’s Modified Eagle Medium supplemented with 10% fetal bovine serum, 100 U/mL penicillin, and 100 µg/mL streptomycin (all reagents from Gibco, Carlsbad, CA, USA). Cells were cultured in a humidified atmosphere at 37 °C with 5% CO_2_.

### 2.2 Isolation and purification of PDEVs

Pollen was suspended in phosphate-buffered saline (PBS) at a 1:40 (w/v) ratio and agitated on an orbital shaker at 180 rpm for 12 hours at 25°C. The pollen suspension was sequentially centrifuged at 4°C to remove cellular debris and large particles:300 × g for 10 minutes,2,000 × g for 20 minutes,10,000 × g for 30 minutes. The supernatant from each step was retained, pooled, and subjected to ultracentrifugation at 100,000 × g for 2 hours at 4 °C to pellet PDEVs. The resulting supernatant was discarded, and the PDEVs-containing pellet was resuspended in PBS. For further purification, the resuspended PDEVs were layered onto a discontinuous sucrose gradient composed of 15%/30%/45%/60% (w/v) sucrose solutions and ultracentrifuged at 100,000 × g for 16 hours at 4 °C. PDEVs were collected from the interface between the 30% and 45% sucrose layers. To remove residual sucrose, the PDEV suspension was diluted with PBS and subjected to a final ultracentrifugation step at 100,000 × g for 2 hours at 4 °C. The purified PDEV pellet was resuspended in PBS and filtered twice through a 0.22μm sterile filter. The final PDEV solution was aliquoted into sterile microtubes and stored at −80 °C until further use.

### 2.3 Quantitative real−time PCR

Total RNA was extracted from cells or tissues using TRIzol reagent (Invitrogen, CA, USA) following the manufacturer’s protocol. cDNA was synthesized from 1 µg of RNA using a cDNA Synthesis Kit (Servicebio, Cat# G3337-100) according to the manufacturer’s instructions. The resulting cDNA was diluted 5- to 10-fold with DEPC and stored at −20 °C for subsequent analysis. qRT-PCR was performed using SYBR Green Master Mix (Servicebio, Cat# G3326-05) on an ABI Prism 7500 Sequence Detection System (Applied Biosystems, USA) as described in the manufacturer’s guidelines. Gene expression levels were normalized to the internal control GAPDH, and relative mRNA abundance was calculated using the 2^−ΔΔCt method. Primer sequences are listed in **Supplementary Table 1**.

### 2.4 Enzyme-linked immunosorbent assay

Lung tissues were homogenized in 1 mL of lysis buffer containing RIPA Lysis Buffer (Beyotime, Cat# P0013B), protease inhibitor cocktail, and phosphatase inhibitor mixture (NCM Biotech, Cat# P002) using a vortex mixer. The homogenate was centrifuged at 12,000 × g for 5 minutes at 4°C, and the supernatant was collected for subsequent ELISA. The levels of IL-4, IL-5, IL-13 and IL-33 were measured with sandwich ELISA kit according to the manufacturer’s instructions (Multi Sciences, China, Cat# EK204HS, EK205HS, EK213, EK233HS). All ELISA absorbance values were determined at 450 nm with a microplate reader.

Total and allergen-specific IgE levels were measured using enzyme-linked immunosorbent assay (ELISA). Total IgE was quantified using the ELISA MAX™ Standard Set Mouse IgE kit (BioLegend) according to the manufacturer’s instructions, with results expressed in μg/mL.

To quantify allergen-specific IgE, 96-well microplates were coated with 100 μL/well of coating solution containing *Artemisia annua* pollen allergen for overnight incubation at 4 °C. On day 2, plates were washed three times with 1× TBST buffer. Blocking was performed by adding 200 μL/well of 3% (w/v) bovine serum albumin (BSA, Sigma-Aldrich, USA) in TBST, followed by 1-hour incubation at room temperature(RT) with orbital shaking at 300 rpm. After three wash cycles, 100 μL of mouse serum samples diluted 1:10 in assay diluent (1% BSA in TBST) were added to respective wells, and plates were incubated overnight at 4 °C.On day 3, following three washes with TBST, horseradish peroxidase-conjugated goat anti-mouse IgE secondary antibody (Thermo Fisher Scientific, USA; Cat# SA5-10263) was diluted 1:1,000 in TBST and applied at 100 μL/well. Plates were incubated for 1 hour at RT in the dark. After three final washes, 100 μL/well of TMB substrate solution (BioLegend, USA) was added, and color development was allowed for 30 minutes at RT in darkness. The enzymatic reaction was terminated by adding 100 μL/well of 2.5 M sulfuric acid, and optical density measurements at 405 nm were recorded using Chromate 4300 microplate reader (Awareness Technologies, USA).

### 2.5 Flow cytometry of bronchoalveolar lavage fluid cells

The flow cytometry methodology was in accordance with the guideline (15). BALF cells were centrifuged at 500 × g for 10 minutes at 4 °C, following the supernatant was collected for other experiments. The cell pellet was resuspended in 500 μL PBS, and the total cell was measured. This centrifugation-resuspension procedure was repeated prior to staining. For surface antigen labeling, cell suspensions were incubated with antibody cocktails for 30 minutes at 4 °C in the dark. Purified rat anti-mouse CD11b-PerCP/Cyanine5.5, anti-CD45-FITC, anti-GR-1-BV421, anti-CD11c-APC, and anti-CD170-PE were purchased from BioLegend or BD Biosciences. Flow cytometric acquisition was performed using a BD LSRFortessa™ X-20 Flow Cytometer, and data analysis was conducted with FlowJo Software version 10.9.0 (BD Biosciences).

### 2.6 Lung histology

Mouse lung tissues were fixed at room temperature in a 4% formaldehyde solution. Subsequently, the tissues were paraffin-embedded, sectioned, and subjected to hematoxylin and eosin (HE) staining and periodic acid-Schiff (PAS) staining. Histopathological images were captured using NDP.view software (version 2.0, Hamamatsu Photonics). Pulmonary inflammation scores were determined through a blinded histological assessment as described previously (16).

### 2.7 Cell viability assay

Cells were seeded into 96-well plates and exposed to serial dilutions of PDEVs for a 24-hour incubation period. Cell viability was subsequently assessed using the cell counting kit-8 (CCK-8) according to the manufacturer’s instructions(Beyotime Biotechnology).

### 2.8 Statistical analysis

We conducted statistical analysis with GraphPad Prism version 8.0 software(GraphPad, San Diego, CA, USA). The values were presented as the means ± standard error of the mean (SEM). P values were calculated with student’s t tests (independent two-sample). *P* value < 0.05 was considered statistically significant. (ns *p*>0.05,*p < 0.05, **p < 0.01, ***p < 0.001, ****p < 0.0001).

## 3 Results

### 3.1 Purification and characterization of PDEVs

**Figure 1:**
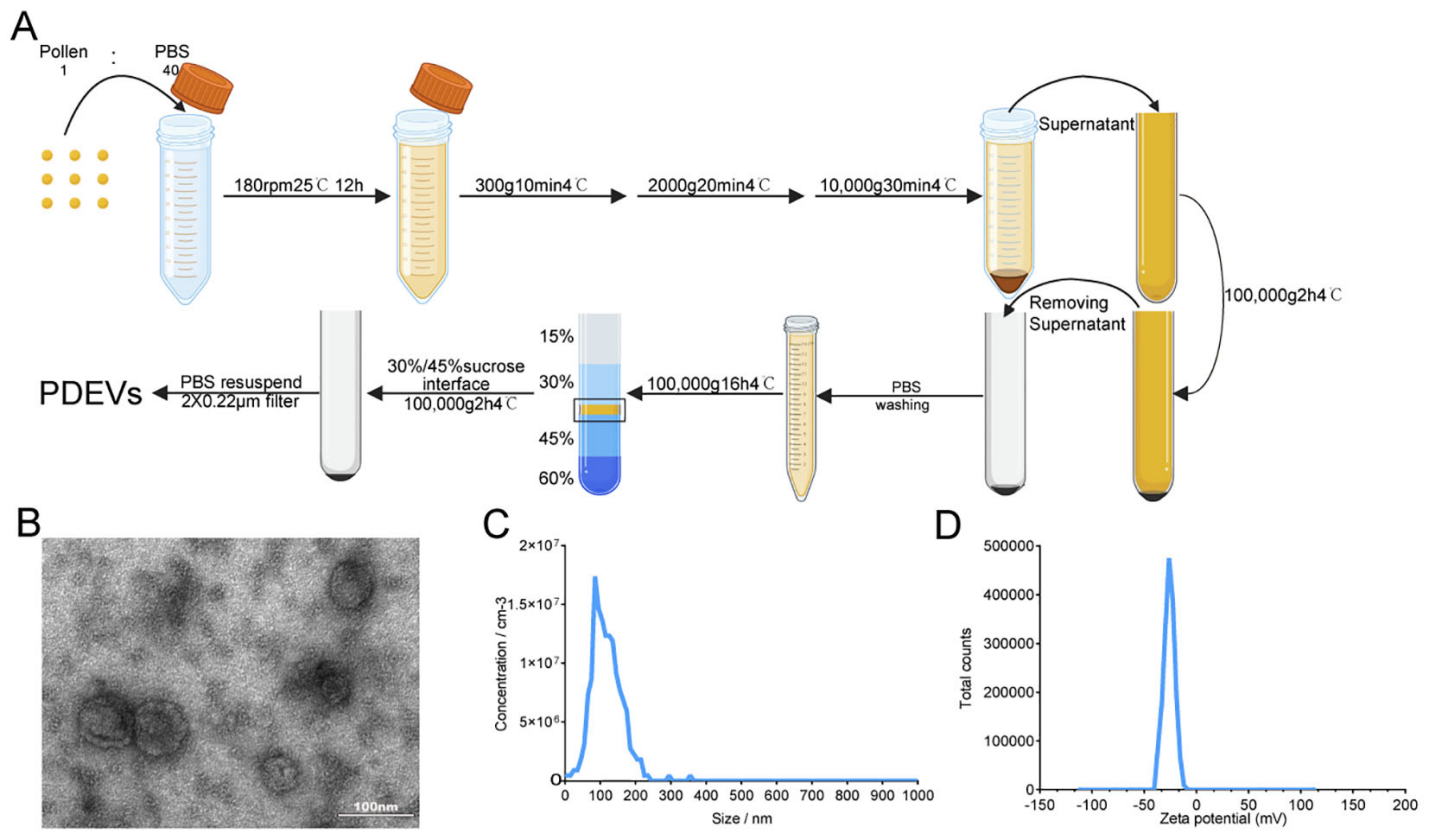
Purification and characterization of PDEVs. **(A)** PDEVs were separated and concentrated by differential centrifugation and sucrose gradient ultracentrifugation. **(B)** PDEVs isolated from the sucrose density gradient (30%/45% sucrose interface) were characterized by TEM (transmission electron microscopy, scale bar=100 nm). **(C)** Particle size and concentration distribution of PDEVs were measured by NanoSight NS 300 system. **(D)** Surface charge was measured via a Zetasizer.

To investigate the presence of EVs in *Artemisia annua* pollen, we isolated and purified exosome-like vesicles from the pollen using a method combining differential centrifugation and sucrose density gradient ultracentrifugation. After sucrose density gradient centrifugation, the bands were found to aggregate between the 30% and 45% interfaces (**Figure 1A**). Transmission electron microscopy(TEM) revealed that the vesicles were spherical in shape and possessed a bilayer membrane structure(**Figure 1B**). The NanoSight NS 300 system analysis determined that the average particle size of PDEVs was about 118 nm, and the concentration of the solution was approximately 1.4*10^11^ vesicles per milliliter(**Figure 1C**). The Zeta potential analysis indicated that PDEVs was -26 mV. The bicinchoninic acid assay(BCA) test quantified that the protein concentration of PDEVs was 378 µg/ml. These results indicate that the pollen contains vesicle-like structures that encapsulate a large amount of proteins.

### 3.2 PDEVs enhance inflammatory cytokine release *in vitro*

**Figure 2:**
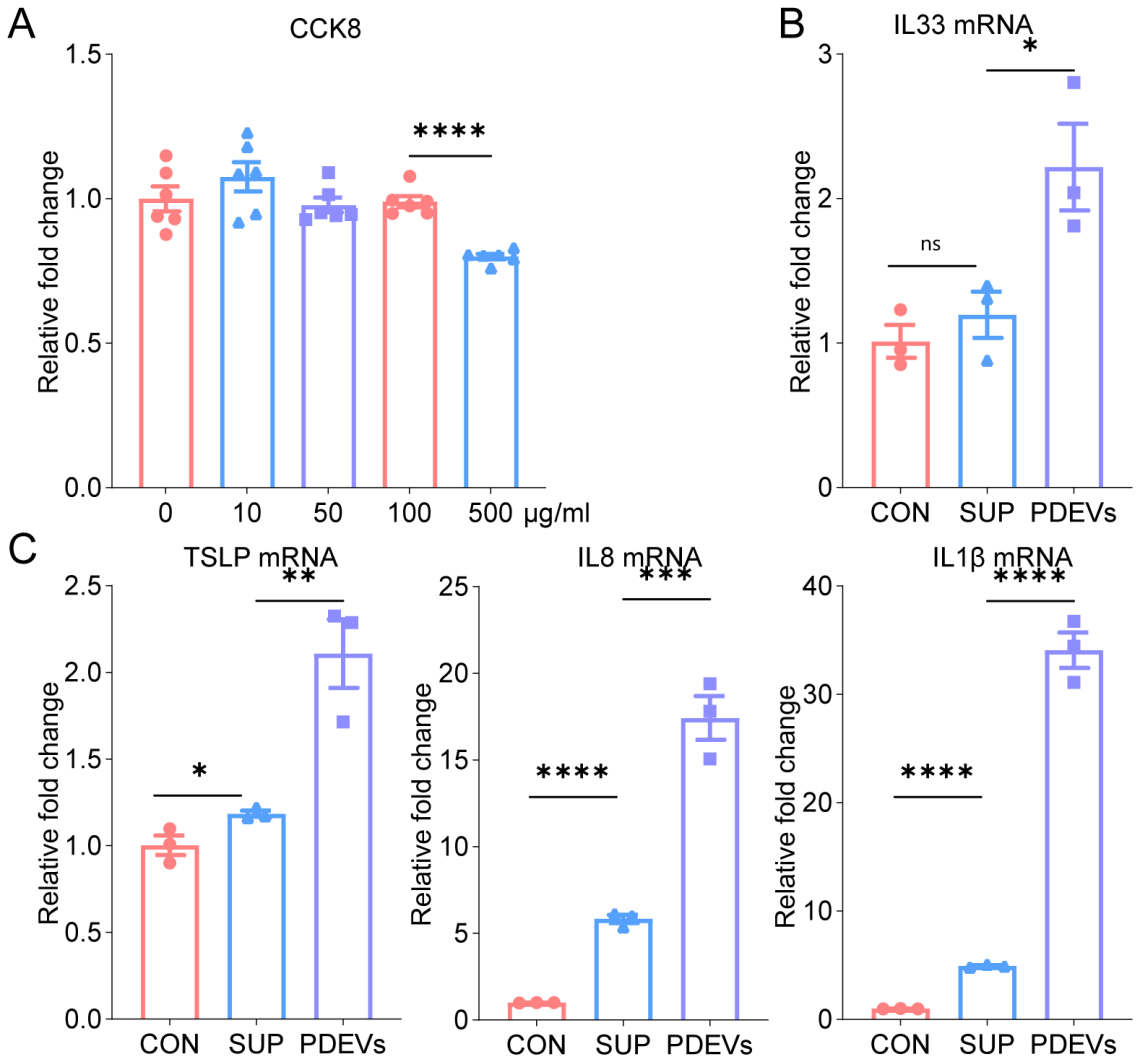
PDEVs enhance inflammatory cytokine release *in vitro*. **(A)** CCK8 analysis of various concentrations of PDEVs on BEAS-2B cell viability(n=6). **(B, C)** qPCR analysis of IL33,TSLP, IL8 and IL1β levels in BEAS-2B cells that were pre-incubated with 50µg/mL PDEVs or SUP(n=3). ns *p*>0.05,*p < 0.05, **p < 0.01, ***p < 0.001, ****p < 0.0001(Student’s t tests). The results are representative data from one of three independent experiments. Shown are representative images, and the data are presented as means ± SEM. ns means non-significant.

Based on previous studies (17, 18), allergenic proteins in Artemisia pollen can stimulate pulmonary epithelial cells to release pro-inflammatory mediators including IL-33, TSLP, and IL-8, which contribute to the pathogenesis of allergic asthma. To evaluate the effects of varying PDEVs concentrations on cell viability, the CCK-8 assay was employed. This assay is based on the activity of intracellular dehydrogenases, which convert cellular metabolic activity into a quantifiable absorbance signal measured at 450 nm, thereby serving as an indirect indicator of cell viability. The CCK-8 assay revealed that PDEVs had no significant effect on cell viability at protein concentrations below 100 μg/ml. (**Figure 2A**) To evaluate their pro-inflammatory potential, BEAS-2B cells were subjected to 6-hour exposure of PDEVs or Sup, followed by quantitative analysis of cytokine expression profiles. Experimental data revealed that both PDEVs and Sup significantly induced mRNA expression of IL-8, IL-1β, TSLP, and IL-33. Notably, PDEVs exhibited markedly enhanced pro-inflammatory activity compared to Sup at equivalent protein concentrations (**Figures 2B, C**).

### 3.3 PDEVs induce enhanced inflammatory responses *in vivo*

**Figure 3:**
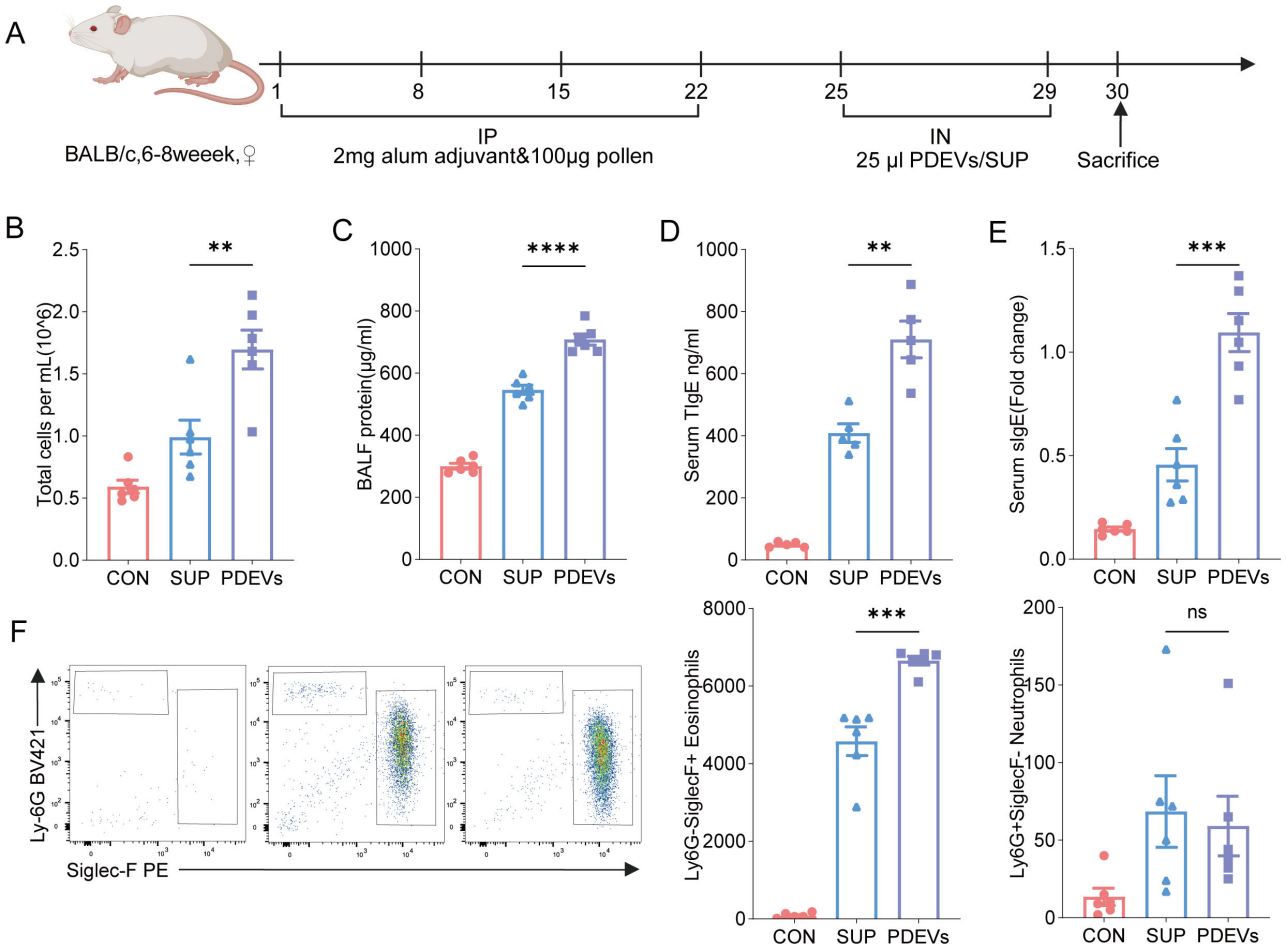
PDEVs induce allergic airway inflammation in mice. **(A)** The simplified experimental scheme. BALB/c mice (n = 6 per group) received intraperitoneal injections(IP) of 2 mg alum adjuvant and 100 μg pollen weekly for four consecutive weeks. Subsequently, mice underwent daily intranasal administration (IN) of 25 μL equal-concentration PDEVs/SUP (25 μg) from days 25 to 29. Mice were sacrificed at day 30. **(B)** Total cell counts per mL in bronchoalveolar lavage fluid (BALF). **(C)** Total protein levels in BALF. **(D)** Serum total immunoglobulin E (TIgE) levels were determined by ELISA and quantified as optical density values at 450 nm. **(E)** Serum specific immunoglobulin E (sIgE) relative levels were determined by ELISA and expressed as optical density values at 450 nm. **(F)** Flow cytometric analysis of frequencies of Ly6G-SiglecF+Eosinophils and Ly6G+SiglecF-Neutrophils in BALF. n = 6 per group. ns *p*>0.05,*p < 0.05, **p < 0.01, ***p < 0.001, ****p < 0.0001(Student’s t tests). The results are representative data from one of three independent experiments. Shown are representative images, and the data are presented as means ± SEM. ns means non-significant.

To further investigate the effects of PDEVs on allergic airway inflammation, we established an asthmatic mouse model through combined intraperitoneal and intranasal administration. Mice were intraperitoneally injected with 100 μg of crude Artemisia annua pollen extract and 2 mg alum adjuvant on days 1, 8, 15, and 22. Subsequently, from days 25 to 29, mice received daily intranasal instillations of 25 μg PDEVs, Sup, or PBS. On day 30, BALF and lung tissues were collected for analysis (**Figure 3A**).

The results demonstrated that both PDEVs and Sup treatments significantly increased total cell counts in BALF compared to PBS controls, with PDEVs inducing a more pronounced elevation.(**Figure 3B**)Notably, PDEVs-treated mice exhibited significantly higher protein concentrations in BALF than Sup-treated mouse.(**Figure 3C**) Meanwhile, serum levels of total IgE(TIgE) and specific IgE(sIgE) exhibited similar results. (**Figures 3D, E**) Flow cytometry analysis revealed that PDEVs significantly enhanced the proportion of eosinophils among CD45+ cells in BALF compared with Sup administration (**Figure 3F**).

**Figure 4:**
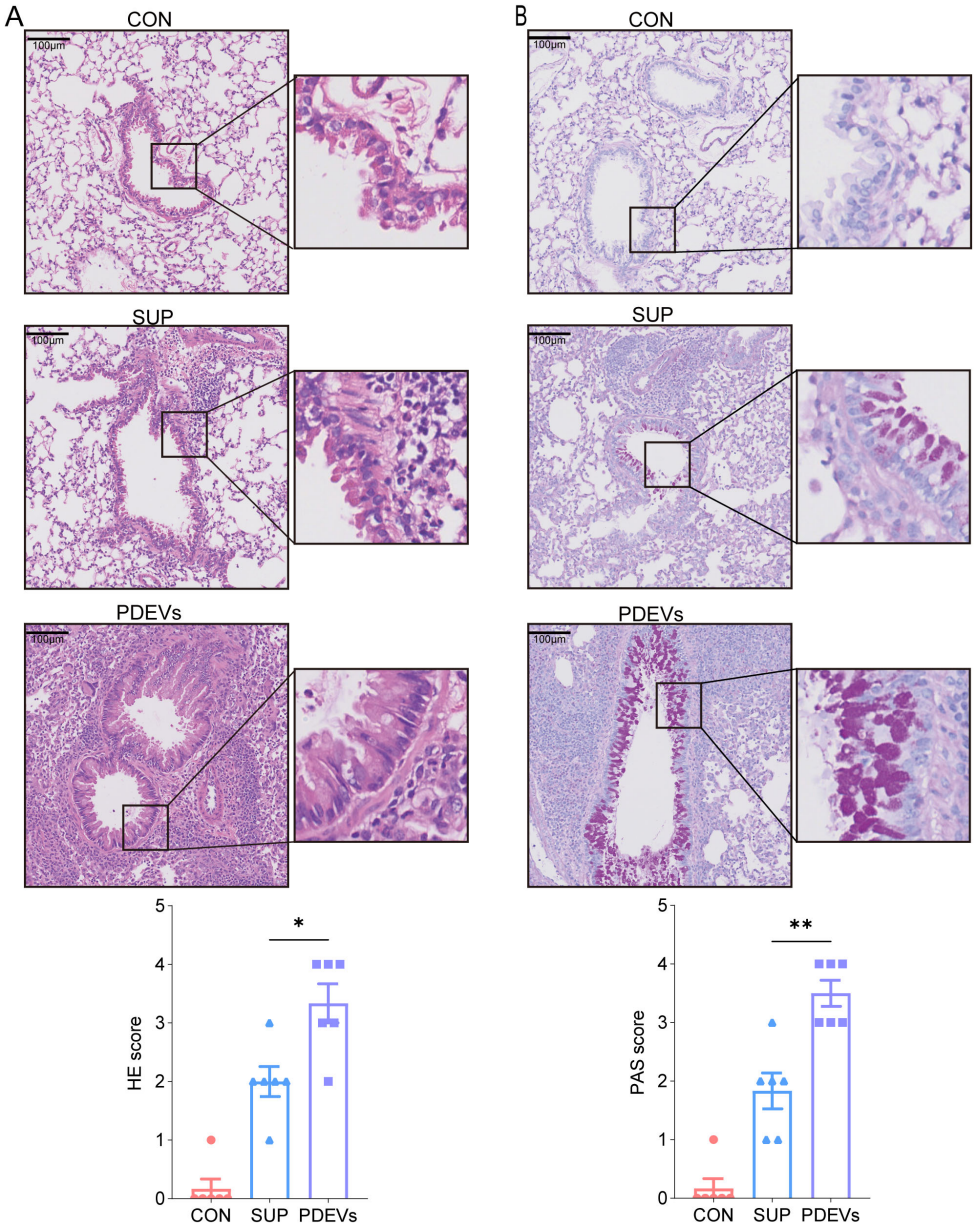
Histological examination of paraffin-embedded lung sections from mice. **(A)** Hematoxylin and eosin (HE) staining of lung tissues and lung inflammation score. Scale bar, 100µm. **(B)** Periodic acid Schiff (PAS) staining of lung tissues and airway mucus secretion score. n = 6 per group. *p < 0.05, **p < 0.01(Student’s t tests). The results are representative data from one of three independent experiments. Shown are representative images, and the data are presented as means ± SEM.

Histopathological evaluation of lung tissues confirmed findings above. HE staining revealed more extensive peribronchial inflammatory cell infiltration in PDEVs-treated mice, accompanied by elevated inflammatory scores. (**Figure 4A**) PAS staining further demonstrated increased mucus obstruction in PDEVs-exposed airways compared to Sup-treated controls (**Figure 4B**).

**Figure 5:**
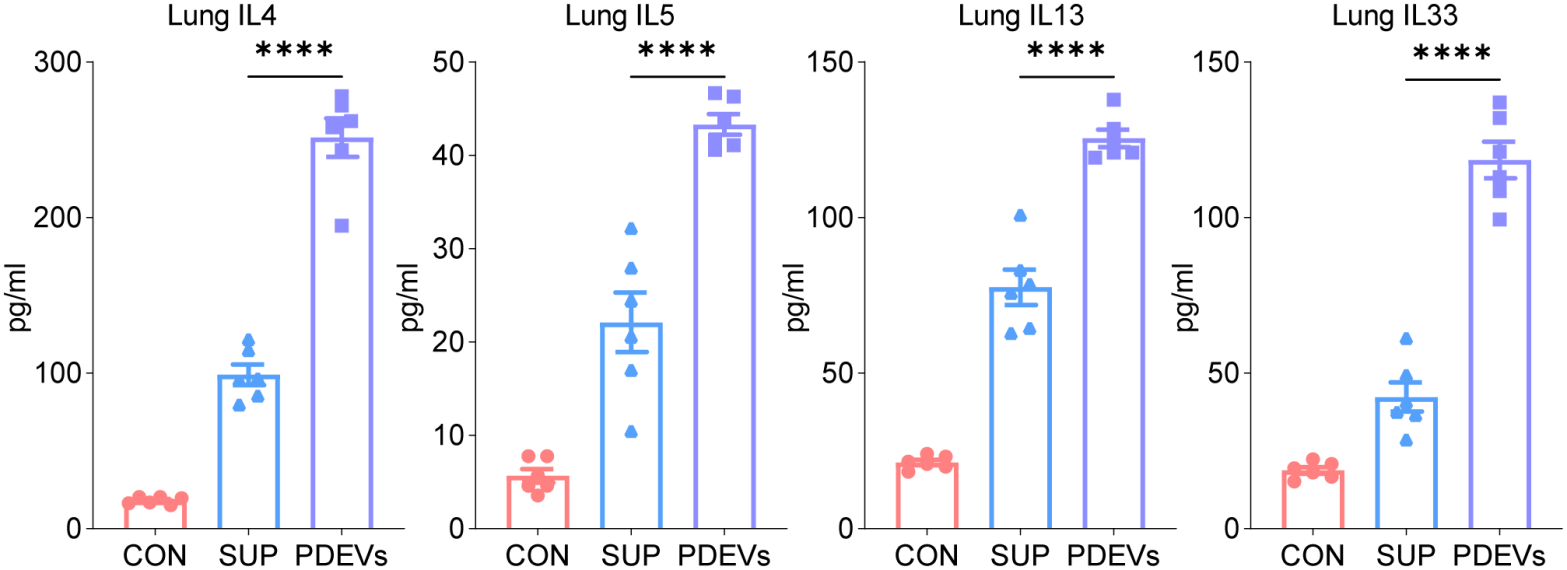
ELISA analysis inflammatory factor in lung of mice. ELISA analysis of IL4, IL5, IL13 and IL33 levels in lung that were pre-incubated with PDEVs or SUP. n = 6 per group. ****p < 0.0001(Student’s t tests). The results are representative data from one of three independent experiments. Shown are representative images, and the data are presented as means ± SEM.

Protein quantification in lung homogenates showed that both PDEVs and Sup enhanced the expression of type 2 cytokines (IL-4, IL-5, IL-13) and IL-33, with PDEVs inducing significantly greater increases than Sup for all measured cytokines (**Figure 5**).

Collectively, these findings indicate that PDEVs exhibit stronger pro-inflammatory activity in experimental asthma models compared to supernatant components.

### 3.4 EVs from other pollen species exhibit similar pro-inflammatory effects *in vitro*

**Figure 6:**
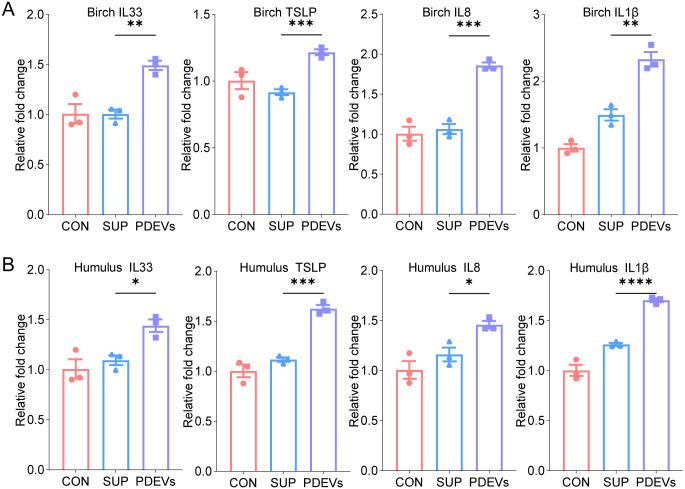
Birch and humulus PDEVs stimulate inflammatory cytokine release *in vitro*. **(A)** qPCR analysis of IL-33, TSLP, IL-8, and IL-1β expression in BEAS-2B cells exposed to 50µg/mL PDEVs, SUP derived from birch pollen. **(B)** qPCR analysis of IL-33, TSLP, IL-8, and IL-1β expression in BEAS-2B cells exposed to 50µg/mL PDEVs, SUP derived from humulus pollen. n = 3 per group. ns *p*>0.05,*p < 0.05, **p < 0.01, ***p < 0.001, ****p < 0.0001(Student’s t tests). The results are representative data from one of three independent experiments. Shown are representative images, and the data are presented as means ± SEM.

To investigate whether EVs exist in other pollen species and exert comparable immunostimulatory effects, we isolated EVs from birch and humulus pollen using the protocol described in **Figure 1A**. Following *in vitro* stimulation of BEAS-2B cells at equivalent protein concentrations, qPCR analysis revealed that PDEVs from both species significantly upregulated the expression of IL33, TSLP, IL8, and IL1β compared to their respective Sup (**Figures 6A, B**). These findings demonstrate that the enhanced pro-inflammatory activity of pollen-derived EVs is not unique to *Artemisia annua* but is common across distinct pollens.

## 4 Discussion

Our study provides compelling evidence that PDEVs represent a critical role in pollen-induced allergic airway inflammation. We have successfully isolated and characterized PDEVs from Artemisia annua pollen, demonstrating that these nanoscale structures exhibit significantly stronger pro-inflammatory activity than pollen supernatant in both *in vitro* and *in vivo* models. Notably, EVs isolated from birch and humulus pollen also demonstrated enhanced inflammatory potential compared to their respective Sup, suggesting a common mechanism across diverse pollen species.

The size of airborne particulate matter significantly influences its biological effects. Particles larger than 10 μm are generally considered incapable of reaching the bronchi, whereas smaller particles can penetrate deeply into the bronchi and alveoli, enabling direct interactions with airway epithelial cells and immune cells (19–21). This deeper deposition may partially explain the enhanced cytokine responses (IL-4, IL-5, IL-13, IL-33) observed in asthma mouse models following exposure to purified particulate matter compared to crude pollen extracts.

Moreover, previous studies on plant-derived extracellular vesicles have demonstrated that their unique structural characteristics, particularly their bilayer membrane architecture, ensure the long-term stability of contents under physiological conditions and protect them from degradation (22–24).

The Zeta potential of -26 mV suggests that PDEVs carry a negative charge, which may facilitate electrostatic interactions with positively charged receptors on epithelial cells or immune cells. This interaction could activate intracellular signaling pathways (e.g., NF-κB, MAPK) that drive the release of pro-inflammatory mediators such as IL-33, TSLP, and IL-8 (25–28). This dual mechanism of action may explain why PDEVs induce stronger eosinophilic infiltration and mucus hypersecretion than pollen extracts alone.

Current research on plant-derived extracellular vesicles has predominantly focused on vesicles isolated from plant leaves and fruits (22, 29). Plant-derived extracellular vesicles have been demonstrated to regulate macrophage polarization, suppress inflammatory responses, and modulate T-cell activation, exhibiting therapeutic potential in various inflammatory conditions including inflammatory bowel disease, psoriasis, diabetes, and colorectal cancer (30). Notably, extracellular vesicles have also been identified in *Artemisia annua* leaves, with several studies confirming their capacity to alleviate acute pulmonary inflammation and regulate the tumor immune microenvironment (31–33).*Artemisia annua* pollen, a prevalent allergen in many regions, is well-established as a potent inducer of allergic airway inflammation. Although previous research has indicated the presence of extracellular vesicles in olive pollen (*Olea europaea*) containing major allergenic proteins (14), there remains a significant knowledge gap regarding the existence of plant-derived extracellular vesicles in *Artemisia annua* pollen. Furthermore, while plant-derived vesicles are generally recognized for their anti-inflammatory properties, the functional role of PDEVs—whether they maintain anti-inflammatory effects or paradoxically contribute to airway inflammation—remains unclear. This study confirms the presence of PDEVs in *Artemisia annua* pollen and reveals a striking contrast to the typical anti-inflammatory profile observed in other plant-derived vesicles. Our findings demonstrate that these PDEVs exhibit significantly enhanced pro-inflammatory activity in airway inflammation models. This unexpected discovery suggests that PDEVs may play a previously unrecognized role in pollen-induced allergic responses, indicating that the immunomodulatory effects of plant vesicles maybe context-dependent and vary by plant tissue source. These insights highlight the importance of considering PDEVs as potential contributors to allergic airway inflammation, which could inform future therapeutic strategies targeting pollen-related respiratory disorders.

Thunderstorm asthma (TA) is significantly correlated with elevated airborne pollen concentrations (34). While traditional hypotheses focus on mechanical pollen rupture during thunderstorms releasing respirable allergen fragments (1–5 μm) (6, 35, 36), our data propose an alternative mechanism: PDEVs serve as pre-formed, biologically active allergen reservoirs that are rapidly mobilized under specific environmental conditions. However, this remains a preliminary hypothesis that requires further validation through quantification of vesicle release under varying humidity conditions and environmental simulation experiments.

While our findings demonstrate the pro-inflammatory potential of PDEVs in both *in vitro* and *in vivo* models, several limitations necessitate further investigation. First, although we observed allergenic inflammation induction, the underlying molecular mechanisms remain to be elucidated. Second, the PBS-based extraction method used for PDEV isolation may not fully recapitulate natural atmospheric conditions where pollen grains primarily interact with rainwater, potentially altering vesicle yield and composition compared to environmental exposure scenarios. Third, there is insufficient experimental evidence to rule out contamination of pollen-derived preparations with particulate matter from other sources, such as diesel exhaust particles, among others. Finally, the clinical translatability of our murine asthma model requires validation. Establishing ex vivo cultures of human bronchial epithelial cells with PDEVs or employing advanced airway organoid models may effectively address this translational gap.

In conclusion, this study highlights the underappreciated role of pollen-derived EVs in allergic airway inflammation. By bridging plant biology, immunology, and public health, our findings open new avenues for understanding and combating the growing burden of allergic diseases in a changing climate.

## Data Availability

The original contributions presented in the study are included in the article/**Supplementary Material**. Further inquiries can be directed to the corresponding author.
